# Neurofeedback Treatment of Negative Symptoms in Schizophrenia: Two Case Reports

**DOI:** 10.1007/s10484-018-9417-1

**Published:** 2018-09-28

**Authors:** Khashayar Pazooki, Max Leibetseder, Walter Renner, Gabriel Gougleris, Efsevia Kapsali

**Affiliations:** 1 Group Psylux, Neuroacademy & Traumainstitut Luxembourg, Cabinet de Psychologie & de Sciences de la Psychothérapie Khashayar Pazooki, Luxembourg, 53, route d´Arlon, 8211 Mamer, Luxembourg; 2 Sigmund Freud University Vienna, Vienna, Austria; 3 Pan-European University, Bratislava, Slovakia; 4 Sigmund Freud University Vienna, Vienna, Austria; 5 Medizinisches Versorgungszentrum Halberg/Saarbrücken, Saarbrücken, Germany; 6 University of Saarland, Saarbrücken, Germany

**Keywords:** Schizophrenia, Negative symptoms, Treatment, Neurofeedback, Case study

## Abstract

Negative symptoms of schizophrenia, like diminished emotional expression and a dearth of self-initiated behavior do not respond reliably to anti-psychotic medication or to conventional psychotherapeutic approaches. Starting from evidence on the probable neural basis of such symptoms and on the effectiveness of neurofeedback with other psychological disorders, the present case study applied 20 sessions of EEG neurofeedback to a 45-year-old female and a 30-year-old male, both diagnosed with severe negative symptoms of schizophrenia. In both cases GAF scores were improved significantly and at the end of treatment, both patients did not meet the diagnostic criteria of negative symptomatology any longer. Symptom reduction went along with an obvious improvement of social, interpersonal, and cognitive abilities according to the clinical impression. Detailed data analysis revealed that these improvements went along with corresponding changes of EEG parameters and with distinct patterns and strategies of change in each of the two individuals. The results suggest that EEG neurofeedback should be examined on a larger scale as it offers a promising alternative to existing treatment approaches for negative symptoms in schizophrenia.

## Introduction

### Negative Symptoms in Schizophrenia

DSM-5 (American Psychiatric Association 2013) has conceptualized psychotic disorders along five key features: delusions, hallucinations, disorganized thinking and speech, disturbed motor behavior, and negative symptoms. These are the negative symptoms listed by DSM-5: (1) diminished emotional expression, (2) avolition, (3) alogia, (4) anhedonia, and (5) asociality, with numbers (1) and (2) being especially pronounced.

*“Diminished emotional expression”* is defined as “reductions of the expression of emotion in the face, eye contact, intonation of speech (prosody), and movements of the hand, head and face that normally give an emotional emphasis to speech. *Avolition* is a decrease in motivated self-initiated purposeful activities. The individual may sit for long periods of time and show little interest in participating in work or social activities” (American Psychiatric Association 2013, p. 88).

Gruber et al. (2014) concluded from previous evidence that cognitive dysfunctions and negative symptoms of schizophrenia had a larger effect on long-term impairment of functioning than positive symptoms. Similarly, Kalin et al. (2015) summarized previous findings indicating that negative symptoms have a detrimental impact on social adaptation and social competence in patients with schizophrenia. Negative symptoms also go along with a reduced quality of life (Browne et al. 1996; Packer et al. 1997) and with an increased risk of relapse (Dyck et al. 2014).

### The Neural Basis of Negative Symptoms

Hinkelmann (2002) summarized and confirmed previous findings, according to which negative symptoms in schizophrenia go along with impaired performance in neuropsychological assessments and with “soft signs” of brain pathology in neurological examinations. Pratt et al. (2008) developed a neurobiological model of “hypofrontality” in schizophrenia, i.e., the cognitive, emotional, and behavioral impairments summarized as negative symptoms were attributed to an impaired function of the prefrontal cortex. More recently, a special volume of *European Neuropsychopharmacology* has been dedicated to current findings on the brain pathology associated with negative symptoms in schizophrenia. Millan et al. (2014) summarized these results indicating that negative symptoms can be explained by “a dysfunction of frontocortico-temporal networks […] together with a disruption of cortico-striatal circuits, though other structures are also involved, like the insular and parietal cortices, amygdala and thalamus” (p. 645f.). Thus, negative symptoms cannot be attributed to the dysfunction of a single, circumscribed area of the brain but rather “reflect anomalies of distributed neural networks” (Millan et al. 2014, p. 653).

Similarly, a recent review of magnetic resonance imaging studies by Gruber et al. (2014) pointed to “an important role of glutamate-dopamine interactions within cortico-striato-thalamo-cortical (CSTC) loops, which are modulated by hippocampal and amygdala inputs, in the pathophysiology of schizophrenic disorders” (p. 7).

It may be speculated that negative symptoms, in part at least, can be explained by attention deficits that are caused by the neurobiological alterations outlined above. Attention can be described at the cerebral level as the inhibition of irrelevant information. The activity of the alpha band (i.e., frequencies ranging from 8 to 12 Hz) in EEG has been shown to be closely related to attentional processes (Klimesch 2012) and, depending on the brain site of the alpha activity and its exact frequency range (fast vs. slow alpha), may have an excitatory or inhibitory function. Merrin and Floyd (1992, 1996) found that negative symptoms in schizophrenia are associated with “reduced alpha power and less alpha coherence between hemispheres and between right parietal and frontal regions” (Merrin and Floyd 1996, p. 151).

Based on quantitative EEG (QEEG) assessments, Begić et al. (2011) found increased delta activity over the fronto-temporal and at the parieto-occipital regions and an increased theta activity in the frontal as well as in the parieto-occipital regions in patients with schizophrenia. Interestingly, Harris et al. (1999) found a significant correlation between beta band activity at the frontal site and psychomotor poverty. Other evidence, as reported by Hyun et al. (2011) suggested attentional deficits to be associated with increased theta (4–8 Hz) activity in the fronto-central, temporal, and parieto-occipital lobe of the cortex and with dysregulation of slow cortical potentials (SCP), i.e., the oscillations, on which the EEG is modulated.

Manuseva et al. (2012) examined gender differences in patients with schizophrenia measuring the amplitude, mean frequency and relative power of the different frequency bands by QEEG. As compared to men, female patients had a higher amplitude and relative power of beta-1 and beta-2 activity and their mean theta was significantly lower than in men over left-frontal temporal and parietal regions.

### Conventional Treatment Approaches

According to Millan’s et al. (2014) extensive review, there is only limited evidence for the effectiveness of medication for the reduction of negative symptoms: antipsychotics, antidepressants, stimulants, anxiolytics, and substances addressing anomalies of glutamatergic transmission were examined in numerous clinical trials during the past years, but no clear recommendation for pharmacological treatment of negative symptoms resulted.

A meta-analysis of 168 placebo-controlled studies comprising a total of 6503 participants in the treatment arm and 5815 participants in the placebo arm has shown that second-generation neuroleptics and antidepressants as well as other medication and psychological interventions had statistically significant effects, but did not lead to clinically relevant symptom reduction. First-generation neuroleptics and brain stimulation had no statistically significant effects (Fusar-Poli et al. 2015).

Conversely, Millan et al. (2014) were cautiously optimistic that “hypofrontality” associated with negative symptoms could be reduced by cognitive behavioral and social skills trainings (Elis et al. 2013; Rus-Calafell et al. 2013), by vocational rehabilitation (Bio and Gattaz 2011), or by repetitive transcranial magnetic stimulation (TMS) applied to the dorsolateral prefrontal cortex (cf., Sürmeli 2014).

Repetitive TMS is still under examination, however, and cognitive behavioral as well as related treatments are not accepted equally by all patients. It should also be considered that, at least in some cases, psychological training has to be continued over extensive periods of time. For example, the training program by Wiedl et al. (2014) is based on up to 58 sessions, comprising 2 weekly appointments over half a year, thus putting high demands on the clients’ patience and willingness to cooperate continuously.

### Neurofeedback

In neurofeedback, the cortical activity of the brain is registered by EEG and after data processing the activity of the various frequency bands is displayed on a computer screen. By watching this display the subject receives feedback about his or her cortical activity and learns to control it. Feedback on the screen is given separately for each of the EEG frequency bands that are of interest according to the clinical diagnosis.

For example, the alpha waves are usually associated with alert relaxation, enhanced focus, concentration, and calmness (Evans and Abarbanel 1999) and Alpha enhancement has been used successfully with pain disorders, depression, and post-traumatic stress disorder (Budzynski et al. 2009; Hurt et al. 2014; Vernon 2005). Beta modulation is used for the reduction of inattention, hyperactivity, and impulsivity (Lubar et al. 1995) in the treatment of Attention Deficit and Hyperactivity Disorder (ADHD) (Arns et al. 2009; Heinrich et al. 2007; Monastra et al. 2005). Specific neurofeedback protocols have been developed for the treatment of anxiety (e.g., Hammond 2005), pain (Dursun and Dursun 2014) as well as addiction (Sokhadze et al. 2014). There is consensus among the empirical reports that neurofeedback leads to self-reinforcing training effects on the basis of operant learning and thus the improvements achieved on the symptom level remain stable over time after the training has ended.

### Rationale of the Present Study

No studies specifically addressing negative symptoms in schizophrenia by neurofeedback were found in our literature search. Concerning the treatment of various symptoms in schizophrenia, Sürmeli (2014) reviewed empirical findings regarding the reduction of stress, sleeping problems and general psychopathology and concluded that neurofeedback had been found to be “‘possibly’ […] to ‘probably’ efficacious”. Sürmeli et al. (2012) found a highly significant reduction of positive, negative, and cognitive symptoms by neurofeedback, but their protocol did not address negative symptoms specifically. The effects were satisfactory, but the intervention comprised an average of 58 sessions (range 24–91) per patient. It may be speculated that a neurofeedback protocol especially directed at reducing negative symptoms might be less time consuming, though equally effective. Considering these encouraging results, neurofeedback should be examined as an intervention specifically addressing the reduction of negative symptoms in schizophrenia. This is the aim of the present study.

These were the research questions:

1. Will EEG parameters differ across therapeutic sessions and will they differ between Condition 1 (instruction) and Condition 2 (no instruction)?
2. Will there be patterns of EEG activity which can be identified consistently across sessions?
3. Can EEG parameters be predicted successfully from other EEG parameters assessed in the same session and what is the relationship of EEG parameters between preceding and consecutive sessions?
4. Will the degree of negative symptomatology be reduced in the course of Neurofeedback interventions?

## Method

### Participants

Two patients participated in this study. One patient was female, 45-year old, former bank clerk and in retirement, since over 15 years diagnosed with ICD-10 schizophrenia, residual type (F 20.5). She had been hospitalized 15 times. Currently, positive symptoms had remitted under medication (Abilify 15 mg 1 × 1 and Topamax 2 × 1). The second participant was a 30-year old male, former university student, diagnosed with ICD-10 Schizophrenia, Residual Type (F 20.5) for 7 years. He had been hospitalized three times and also had remitted under medication (Abilify 25 mg 1 × 1). Both patients continued medication during neurofeedback treatment.

Inclusion criteria were (1) a clinical diagnosis of schizophrenia, (2) marked negative symptoms, and (3) good compliance. From the participants of an inpatient psychiatric rehabilitation program at the Medizinisches Versorgungszentrum Halberg/Saarbrücken, Germany, who met these criteria, the two participants were selected at random by their psychiatrist at the clinic (i.e., the fourth author of this paper).

The age of initial manifestation was 22 years for the male patient and 23 years for the female patient. The female patient had been hospitalized over five times and the male patient over three times because of acute psychotic symptoms. First negative symptoms were assessed in both patients after the first three hospitalizations. Both patients showed good compliance and blood levels of medication were in the effective range. The female patient had worked in a bank as a trained banker until 2012. The male patient had terminated his post-high school education due to the illness and was not able to pursue a regular employment any longer due to his negative symptoms. Social contact was reduced in both patients but both of them have been integrated well into their families.

Both patients participated in an inpatient psychiatric rehabilitation program, including psychoeducation, ergo-therapy, and occupational therapeutic interventions. A training for work (low-level work under 3 h) was recommended, but neither of the two patients was able to participate. As a result, ergo-therapeutic measures took place once or twice a week instead, including cognitive training and psychiatric treatment took place every 4 weeks. These were the initial conditions before commencement of neurofeedback treatment.

### Procedures

*Phase 1*, i.e., baseline assessment of the dependent variables was carried out in the course of 1 week.

*Phase 2* was the first intervention period and lasted for 2 weeks with the basic protocol, the participants receiving detailed instructions (i.e., at the beginning of each session, the participants were instructed to stay relaxed while focusing their attention on the computer screen, avoiding distraction). We used Mind-Media Nexus-10 (model MK-II) devices for neurofeedback training, with shielded EXG cables and 250 Hz sampling rate. Artifacts were managed manually; EMG artifacts were inhibited during each training at a maximum of 1.5 µV and feedbacked on the screen with a red bar-diagram for patients to remain calm and stay underneath. Any EMG artifact above 1.5 µV would stop the animation (puzzles) automatically and make the patients regulate their EMGs. We augmented SMR (12–15 Hz) at the contra-lateral side to the handedness of the participants, i.e., C4 for the right-handed female and C3 for the left-handed male participant. Four to eight hertz theta was inhibited at the same electrode site.

*Phase 3* was the second phase of intervention over 2 weeks with a modified protocol: the participants did not receive specific instructions (like the reminder to stay focused in Phase 2) but were advised to continue the training on their own. Although refraining from giving instructions, the therapist still was present during the entire sessions. During the third phase, we again augmented 12–15 Hz SMR at the contra-lateral side to the handedness of both participants and inhibited 4–8 Hz theta at the same electrode site. Additionally, we augmented beta-I (13–18 Hz) at F3 electrode site for both patients and 4–8 Hz theta was reduced at this electrode site. Continuous visual feedback was used for all sessions. Both patients could see their own SMR, Theta, and Beta activities on the screen in form of bars/diagrams. EEG data, such as SMR activity, theta and beta as well as the EMGs were measured throughout each training over 5 min baseline and 30 min effective training time. EEG data was analyzed by mean amplitude of each frequency band that was trained and compared over 20 sessions.

*Phase 4* was a follow-up period, lasting for 1 week, with no interventions taking place. Again, all the variables were assessed.

Sessions lasted for 35 min each, consisting of 5 min baseline and 30 min effective training time.

### Measures

The following EEG parameters were assessed:

- *Mean θ* The amplitude average of theta in µV
- *Mean α* The amplitude average of alpha in µV
- *Mean SMR* The amplitude average of SMR in µV
- *Mean β* The amplitude average of beta in µV
- *Mean EMG* The amplitude average of the artefacts in V.

#### *CompACT-SR* (Prieler 2011)

This is a computerized assessment of reaction time, alertness and selective attention under go/no-go conditions. Either auditory stimulation with prior notice, auditory stimulation without prior notice, or visual stimulation with or without prior notice can be used.

#### Global Assessment of Functioning (GAF)

This is a numeric rating scale assessing the social, occupational and psychological functioning of an individual used by mental health clinicians (Hall 1995). The *GAF* scale was measured by Dr. med. Gavriil Gougleris (neuro-psychiatrist) in phase one and phase four. The inter-rater reliability was calculated by grouping the scores into 5-point intervals (Haro et al. 2003).

#### Positive and Negative Symptom Scale (PANSS)

Negative symptoms were assessed by the German version of the Positive and Negative Syndrome Scale (Kay et al. 2011; Stanley et al. 1987).

### Data Analysis

The neurofeedback sessions one through 20 (Variable A) and Condition 1 (with instruction) vs. Condition 2 (without instruction) (Variable B) served as the independent variables.

Research question (1) was analyzed by Shine’s (1973) two-factorial analysis of variance for single-subject designs. Research question (2) was examined by cluster analyses (Ward algorithm). Part 1 of research question (3) was examined by multiple regressions. In order to assess the effects of two preceding sessions on the consecutive session [part 2 of research question (3)] autocorrelations were computed. Finally, research question (4) was examined by computing Reliable Change Indices (Jacobson and Truax 1991) for each of the assessment tools.

## Results

- Research question (1): differences of EEG parameters between sessions (A) and between conditions “instruction vs. no instruction” (B)? (Pazooki 2018).

Participant K. T.

The results for variable Mean θ were: condition A (η = 0.02; z = 3.48; p = 0.01); condition B [(F1; 5) = 1.87; n. s.] and for the interaction AB [(F 1; 9), p = 0.71; n. s.].

For Mean α: condition A (η = 0.12; z = 3.31; p = 0.01), condition B [(F1; 5) = 1.52; n. s.]; interaction AB [(F 1; 9), p = 0.54; n. s.].

For the variable Mean SMR results were: condition A (η = 0.08; z = 3.17; p = 0.01), B [(F1; 5) = 1.30; n. s.], interaction AB [(F 1; 9) = 0.98; n. s.].

For variable Mean β the results were: condition A (η = 0.09; z = 3.34; p = 0.01), condition B [(F1; 5) = 2.52; n. s.]; interaction AB [(F 1; 9) p = 0.66; n. s.].

For Mean EMG the results were: condition A (η = 0.13; z = 3.29; p = .001), for condition B [(F1; 5) = 0.95; n. s.] and for the interaction AB [(F 1; 9) p = 0.54; n. s.].

Participant B. Z.

For the variable Mean θ: condition A (η = 0.15; z = 3,26; p = 0.01); condition B [(F1; 5) = 0.58; n. s.]; interaction AB [(F 1; 9) = 0.99; n. s.].

For Mean α: condition A (η = 0.32; z = 2.96; p = 0.01), condition B [(F1; 5) p = 0.53; n. s.]; interaction AB [(F 1; 9) = 3.22; 0.05 < p < 0.10].

For Mean SMR: condition A (η = 0.38; z = 2.85; p = 0.01), condition B [(F1; 5) = 0.37; n. s.]; interaction AB [(F 1; 9) = 2.14; n. s.].

For Mean β: condition A (η = 0.25; z = 3.06; p = 0.01), condition B [(F1; 5) p = 0.53; n. s.], interaction AB [(F 1; 9) p = 0.58; n. s.].

For Mean EMG: condition A (η = 0.23; z = 3.13; p = 0.01), condition B [(F1; 5) = 1.26; n. s.] interaction AB [(F 1; 9) p = 0.63; n. s.].

The critical values (Bortz 1977) are as follows: F(1; 5)_5 %_ = 6,61 and F(1; 5)_10%_ = 4,01. F(1; 9)_5%_ = 5,12 and F(1; 9)_10%_ = 3.36.

It can be seen that for both participants and or all the EEG and EMG means, parameter differences across sessions were significant, i.e., in the course of the neurofeedback training, these means have changed. It is also evident that, for both participants and for all the variables assessed, there were no significant differences between the condition with instruction and the condition without instruction, i.e., the brain activities learned in the course of the training with instruction could be transferred successfully to situations were instructions were not present any longer.

- Research question 2: consistent patterns of EEG parameters across sections^1^

For participant K. T., cluster analysis yielded two clusters as follows:

Cluster 1: Mean θ

Cluster 2: Mean α, Mean SMR, Mean β and Mean EMG.

Whereas the single variable in Cluster 1 represents dissociation, Cluster 2 represents tension vs. relaxation. Cluster 1, however, consists of an “outlier variable” (cf., Backhaus et al. 2003, p. 528), suggesting a second cluster analysis without Mean θ, which resulted in the following two clusters:

Cluster 1: Mean α, Mean β

Cluster 2: Mean SMR, Mean EMG.

Whereas Cluster 1 variables express different levels of attention, Cluster 2 variables mainly pertain to the brain’s sensorimotor functions and muscular innervations.

For participant B. Z., the following results were obtained from Cluster Analysis:

Cluster 1: Mean θ, Mean α

Cluster 2: Mean SMR, Mean EMG.

Again, Cluster 1 variables address attentional issues, theta being associated with dissociation and alpha with a relaxed, joyful state on mind, whereas Cluster 2 variables rather pertain to physiological functions than to psychological states of attention.

Thus, in both cases, meaningful results were obtained from Cluster Analyses. Obviously, however, there are individual differences, which—as far as the present single case design is concerned—also could have occurred at random.

- Research question (3): the relationship of EEG parameters within sessions and across sessions

Mean EEG parameters were predicted by other Mean EEG parameters *within* the same sessions by multiple regression. The results obtained from Participant K. T. are presented in Table 1, indicating a large degree of interdependence of EEG variables during the therapeutic process.

**Table 1:** Predictors of EEG parameters from previous sessions (participant K. T.)

Criterion: Mean θ	
Predictors: Mean α, Mean SMR, Mean β, Mean EMG	
Model: R = 0.988; sum of squares = 321.029; df = 4; 15. Mean of squares = 80,257; F = 154.039; p = 0.000	
Mean α	β = 1.341; T = 5.452; p = 0.000
Mean SMR	β = − 0.542; T = − 1.431; p = 0.173
Mean β	β = − 0.077; T = − 0.558; p = 0.585
Mean EMG	β = 0.253; T = 1.130; p = 0.276

Criterion: Mean α	
Predictors: Mean θ, Mean SMR, Mean β, Mean EMG	
Model: R = 0.995; sum of squares = 43.771: df = 4; 15. Mean of squares = 19.954; F = 423.178; p = 0.000	
Mean θ	β = − 0.496; T = 5.452; p = 0.000
Mean SMR	β = 0.725; T = 4.959; p = 0.000
Mean β	β = 0.103; T = 1.284; p = 0.219
Mean EMG	β = − 0.333; T = − 2.951; p = 0.010

Criterion: Mean SMR	
Predictors: Mean θ, Mean α, Mean β, Mean EMG	
Model: R = 0.958; sum of squares = 67.130; df = 4; 15. Mean of squares = 6.200; F = 357.124; p = 0.000	
Mean θ	β = − 0.229; T = − 1.431; p = 0.173
Mean α	β = 0.857; T = 4.950; p = 0.000
Mean β	β = − 103; T = − 1.165; p = 0.262
Mean EMG	β = 0.490; T = 5.557; p = 0.000

Criterion: Mean β	
Predictors: Mean θ, Mean α; Mean SMR; Mean EMG	
Model: R = 0.958; sum of squares = 67.130; df = 4; 15. Mean of squares = 16.783; F = 164.513; p = 0.000	
Mean θ	β = − 0.265; T = − 0.558; p = 0.585
Mean α	β = 0.962; T = 1.284, p = 0.219
Mean SMR	β= − 0.810; T = − 1.165; p = 0.262
Mean EMG	β = 1.099; T = 3.3555; p = 0.004

Criterion: Mean EMG	
Predictors: Mean θ, Mean α, Mean SMR; Mean β	
Model: R = 0.985; sum of squares = 238.209; df = 4;15. Mean of squares = 59.552; F = 125.062; p = 0.000	
Mean θ	β = 0.310; T = 1.130; p = .276
Mean α	β = − 1.103; T = − 2.951; p = 0.010
Mean SMR	β = 1.373; T = 5.557; p = 0.000
Mean β	β = 0.390; T = 3.355; p = 0.004

For Participant K. T., mean EEG parameters were predicted by other mean EEG parameters from previous sessions by multiple regression. Thus, the results indicate a large degree of interdependence of EEG variables in the course of therapy. For participant B. Z., on the other hand, only insignificant results were obtained from the regression analyses.

In order to examine the relationship of EEG parameters *across* sessions, autocorrelations were computed with lag = 1 and lag = 2 and tested for significance by the Box–Ljung statistic (= value). The results for both participants are presented in Table 2 (Pazooki 2018, p. 60).

**Table 2:** Autocorrelations

	Participant K. T.			Participant B. Z.		
	Auto-correlation	Value	Sig	Auto-correlation	Value	Sig
Mean θ						
Lag = 1 (df = 1)	− 0.311	2.145	0.143	− 0.457	4.632	0.031
Lag = 2 (df = 2)	− 0.094	2.353	0.308	− 0.159	5.224	0.073
Mean α						
Lag = 1 (df = 1)	− 0.288	1.838	0.175	− 0.239	1.270	0.260
Lag = 2 (df = 2)	0.062	1.929	0.381	0.133	1.683	0.431
Mean SMR						
Lag = 1 (df = 1)	− 0.233	1.200	0.273	− 0.495	5.427	0.020
Lag = 2 (df = 2)	0.052	1.262	0.532	0.038	5.452	0.065
Mean β						
Lag = 1 (df = 1)	− 0.380	3.203	0.073	− 0.518	5.948	0.015
Lag = 2 (df = 2)	0.041	3.243	0.198	− 0.038	5.973	0.050
Mean EMG						
Lag = 1 (df = 1)	− 0.242	1.299	0.245	− 0.620	8.534	0.003
Lag = 2 (df = 2)	− 0.030	1.320	0.517	0.396	12.210	0.003

In the case of Participant K. T., none of the autocorrelations was significant. Conversely, for Participant B. Z., most autocorrelations were significant. This was the case both, for a lag = 1 and a lag = 2. For the variable Mean EMG there was a significant autocorrelation of − 0.377 on lag 3 (value = 15.701; df = 3; p = 0.001). Thus, for B. Z., EEG parameters of consecutive sessions were highly dependent on EEG parameters of preceding sessions, whereas this was not the case for K. T.

From these results it can be seen that the two participants have employed different strategies of change: Participant K. T. developed distinct patterns of change in each session separately. Thus, she oriented by the current differences between the current EEG variables, but did not orient by the changes from previous sessions.

In contrast, Participant B. Z., coordinated each EEG variable in each session separately. Rather than orienting by the differences between current EEG variables, he oriented by the changes of each individual EEG variable across the previous sessions.

- Research question (4): reduced negative symptoms

In order to investigate improvements of cognitive functions and negative symptoms, the results from the Go/No-Go task, GAF values, and the categorical criteria derived from the PANSS were compared pre- and post-therapy. Differences between the two measurement occasions were examined by critical differences as well as the Reliable Change Index (RCI, Jacobson and Truax 1991). Taking the explorative nature of the study into account, a significance level of p = 0.10 was tolerated, i.e., statistical trends were interpreted as meaningful results (cf., Huber 1973).

1. For participant T. K., reaction times with the Go/No-go task and auditory stimulation were 309 ms pre and 266 ms post treatment (RCI = 3.45). Reaction times for participant B. Z. were 407 ms pre and 386 ms post treatment (RCI = 5.70). For both participants, a statistical trend indicated improvements between pre and post treatment measurements.
2. For subject T.K., the GAF values were: GAF = 51 (z = 0.1) before and GAF = 61 (z = 1.1) after treatment. For subject B.Z., the values were GAF = 55 (z = 0.5) before and GAF = 65 (z = 1.5) after treatment. Assuming a reliability of r_tt_ = 0.87, the RCI = 5.56. The GAF values before and after therapy significantly differ for both subjects.
3. In addition, negative symptoms were assessed categorically by the PANSS before and after the interventions. Before the intervention, both participants, K. T. and B. Z. fulfilled the diagnostic criteria of negative symptomatology, whereas after the intervention none of the two participants met these criteria.

## Discussion

We found that EEG parameters differed significantly in the course of the interventions with both participants who had also successfully transferred their training effects from a condition with instruction to a condition without instruction. Thus, both participants in the course of treatment showed significant learning effects with regard to the EEG parameters involved.

We also found that the patterns of EEG parameters correlating with each other in each session differed between participants; moreover, each participant had developed his or own personal strategy of change: whereas K. T. oriented the mean amplitude of his (her) EEG frequency bands by the amplitude of concurrent other EEG frequency bands, B. Z. oriented by the amplitudes of the same EEG frequency band from preceding neurofeedback sessions. The negative autocorrelations point towards a reduction of high and an increase of low amplitudes, i.e., in the course of treatment a regression to the mean took place in participant B. Z. Quite clearly the interpretation of individual patterns of change remains highly speculative, however, as long as only two single cases can be discussed.

In line with our expectations, we found significant improvements of negative symptomatology in both participants, both by psychometric measures and by the clinical impression of their social and interpersonal behavior. There was a marked increase in spontaneous verbal behavior accompanied by considerable improvement in sociability as well as motivation towards self-initiated activities. These findings suggest that neurofeedback poses a promising treatment of negative symptoms in schizophrenia and should be examined in more detail with respect to its effectiveness and to the patterns and possible mechanisms of change.

Obvious limitations of this exploratory study pertain to the question whether the results obtained in the two single cases can be generalized and replicated as well as to the fact that no follow-up data were collected. Therefore, systematic replication studies, including follow-up data collection, larger sample sizes and more objective outcome measures in a clinical setting could be a next step towards establishing neurofeedback as a treatment of negative symptoms in schizophrenia.

## Footnotes

1 The detailed results of the cluster analyses will be available from the authors upon request.
